# Prevalence of Malaria Infection and Risk Factors Associated with Anaemia among Pregnant Women in Semiurban Community of Hazaribag, Jharkhand, India

**DOI:** 10.1155/2015/740512

**Published:** 2015-10-13

**Authors:** Mohammad Sohail, Shayan Shakeel, Shweta Kumari, Aakanksha Bharti, Faisal Zahid, Shadab Anwar, Krishn Pratap Singh, Mazahirul Islam, Ajay Kumar Sharma, Sneh Lata, Vahab Ali, Tridibes Adak, Pradeep Das, Mohammad Raziuddin

**Affiliations:** ^1^University Department of Zoology, Faculty of Sciences, Vinoba Bhave University, Hazaribag, Jharkhand 825301, India; ^2^Department of Biotechnology, VIT University, Vellore, India; ^3^Department of Biotechnology, Shri Venkateshwara University, Amroha, India; ^4^Division of Biochemistry, Rajendra Memorial Research Institute of Medical Sciences (ICMR), Agam Kuan, Patna 800007, India; ^5^Medical Biology Department, Deanship of Preparatory Year, Jazan University, Jizan, Saudi Arabia; ^6^Female OPD, Sadar Hospital, Hazaribag, Jharkhand 825301, India; ^7^National Institute of Malaria Research (ICMR), Sector 8, Dawarka, Delhi 110077, India; ^8^Division of Molecular Biology, Rajendra Memorial Research Institute of Medical Sciences (ICMR), Agam Kuan, Patna 800007, India; ^9^Ranchi University, Ranchi, Jharkhand 834001, India

## Abstract

The escalating burden, pathogenesis, and clinical sequel of malaria during pregnancy have combinatorial adverse impact on both mother and foetus that further perplexed the situation of diagnosis, treatment, and prevention. This prompted us to evaluate the status of population at risk of MIP in Hazaribag, Jharkhand, India. Cross-sectional study was conducted over a year at Sadar Hospital, Hazaribag. Malaria was screened using blood smear and/or RDT. Anaemia was defined as haemoglobin concentration. Pretested questionnaires were used to gather sociodemographic, clinical, and obstetrical data. The prevalence of MIP was 5.4% and 4.3% at ANC and DU, and 13.2% malaria was in women without pregnancy. Interestingly, majority were asymptomatically infected with* P. vivax* (over 85%) at ANC and DU. Peripheral parasitemia was significantly associated with fever within past week, rural origin of subjects, and first/second pregnancies in multivariate analysis, with the highest risk factor associated with fever followed by rural residence. Strikingly in cohort, anaemia was prevalent in 86% at ANC as compared to 72% at DU, whereas severe anaemia was 13.6% and 7.8% at ANC and DU. Even more anaemia prevalence was observed in MIP group (88% and 89% at ANC and DU), whereas severe anaemia was 23% and 21%, respectively. In view of observed impact of anaemia, parasitemia and asymptomatic infection of* P. vivax* during pregnancy and delivery suggest prompt diagnosis regardless of symptoms and comprehensive drug regime should be offered to pregnant women in association with existing measures in clinical spectrum of MIP, delivery, and its outcome.

## 1. Introduction

Malaria in tropical regions, which is caused by the protozoan parasites* Plasmodium falciparum* and* Plasmodium vivax*, is responsible for 515 million clinical cases [1] and 1 to 3 million deaths annually [2].* Plasmodium vivax*, the most widespread parasite causing human malaria, is responsible for estimated 130–435 million infections annually and is the major cause of malaria in most of Asia and Latin America [3]. Although* P. vivax* infection is commonly considered to be much more benign than* Plasmodium falciparum* infection, historical evidence suggests significant mortality associated with* P. vivax* malaria in the preantimalarial era [4], and death caused by* P. vivax* malaria has been increasingly recognized over the past few years [3, 5]. Hazaribag, the region under investigation, was primarily dominated by* P. vivax* whereas some buffering, bordering, and adjoining regions have lower prevalence of* P. falciparum* and mixed infection. The other human infecting* Plasmodium* parasites, like* P. ovale*,* P. malariae*, and* P. knowlesi,* are the rarest in Indian isolates and these parasites neither were observed during our investigation nor have been reported previously from Jharkhand.

The emergence and spread of drug resistance to commonly used chemotherapeutics are major factors contributing to this increasing burden and most of the mortality and morbidity are borne by children and pregnant women. Pregnant women and their infants are susceptible to common and preventable infectious diseases including malaria but are woefully left unscreened and untreated. According to an estimate, approximately 125 million pregnant women worldwide are exposed to the risks of malaria in pregnancy (MIP) each year, resulting in 200,000 infant deaths [6]. Every year, in India, 28 million pregnancies take place with 67,000 maternal deaths (Registrar General of India, Sample Registration System, Special Bulletin on Maternal Mortality in India, 2004-06), with 1 million women left with chronic ill health and 1 million neonatal deaths [7]. Pregnancy is an event of immunologic tolerance, whereby a woman accepts the implantation of the fetal allograft in her uterus; initiating a gestation phase becomes physiologically susceptible and vulnerable to malaria infection. Pregnant women with relatively lower levels of previously acquired immunity are particularly at high risk of the most severe complications of malaria during pregnancy, such as cerebral malaria, severe malaria anaemia, abortions, intrauterine fetal death, premature delivery, stillbirths, and maternal and infant mortality [6, 8, 9]. In malaria endemic areas, pregnant women are more susceptible to* Plasmodium* infections than their nonpregnant peers. The adverse outcomes of these infections are primarily felt by primigravidae [10, 11], although, in areas of low or unstable transmission, women of all gravidities may be equally at risk [11]. Pregnant women are 3 times more likely to suffer from severe disease as a result of malarial infection compared with their nonpregnant counterparts and have a mortality rate from severe disease that approaches 50% [12, 13].

In spite of severe and fatal consequences of malaria during pregnancy for the mother, foetus, and newborn child, the harmful effects can be substantially prevented and reduced [14] either by using available interventions or through appropriate treatment upon early and stringent diagnosis [15–17]. Because malaria infection during pregnancy is often asymptomatic, the most common control strategy is intermittent preventive treatment during pregnancy (IPTp), designed to clear any malaria infection present at the time of treatment and also to provide posttreatment prophylaxis to prevent infection for a period of weeks. However, increasing concern of widespread resistance of commonly used antimalarial drugs [18, 19] over the globe has opened the avenues for alternative and effective interventions. The diagnosis of malaria during pregnancy is complicated by several factors, including multistage pregnancy terms lacerated with diminished immunity, increased susceptibility of severe diseases, various obstetric complications, splenic and placental sequestration of parasites, various forms of anaemia, and variation in patient presentation. Thus, development of prompt and accurate diagnosis is an important goal of MIP research.

*P. falciparum* malaria during pregnancy is a well-known cause of maternal and fetal morbidity and mortality. Although* P. vivax *infection has received less attention than* P. falciparum *infection, it is clearly an important contributor to both maternal anaemia and low birth weights [20–23] where they frequently coexist. However, of 50 million pregnancies occurring each year in countries where malaria is endemic, approximately one-half occur in areas where* P. vivax* malaria is endemic [14]. Although* P. vivax* infection during pregnancy has been recognized for many years [20], the impact of such infection during pregnancy has been assessed only recently. In series from Thailand and India, women with* P. vivax* infection were more commonly anaemic and delivered lower birth weight neonates, compared with uninfected women, but the effects were less pronounced than those associated with* P. falciparum* infection [21, 22]. In both studies,* P. vivax* infection was most common during the first pregnancy, and the prevalence of such infection peaked early during the second trimester.

Limited and past MIP studies in India have demonstrated the important contribution of malaria to maternal and neonatal morbidity and mortality [21, 23, 24]. Although preliminary results from earlier studies carried out primarily in central India suggest that both* P. falciparum* and* P. vivax* are associated with adverse pregnancy outcomes, these studies primarily focused on symptomatic pregnant women infected with* vivax* [21, 25]. Relatively little information is available from India about* vivax* associated malaria during pregnancy, particularly from Jharkhand, an understudied and tribal dominant region with perennial malaria transmission zone where malaria is rampant and causing sizable annual malaria deaths, second to Orissa in India as per the latest observations published by Dhingra et al. [26] and Hussain et al. [27], which reflects the importance of the area and its necessity of undertaking extensive investigation in terms of malarial pathology concerned and by Hamer et al. [23] reflecting the malaria during pregnancy associated with an increased risk of neonatal and infant mortality.

Thus, in view of the limited information on asymptomatic and* vivax* infection during pregnancy in India, it prompted us to investigate with an objective to better define the estimate of MIP, the prevalence of asymptomatic malaria, and the relative contribution of* P. falciparum *and* P. vivax *during pregnancy and at delivery. To the best of our knowledge, such profile, epidemiological association, and clinical correlation have not been investigated before on isolates of malaria in pregnancy from Hazaribag, Jharkhand, among malaria endemic regions of India. Most significantly, our investigation will be the first report attempting to evaluate the interplay among anaemia, pregnancy, and asymptomatic malaria, stratified according to clinical groups in adult population residing in a perennial transmission zone with a codominance of* P. vivax* and* P. falciparum* prevalent region. Thestudy was conducted at Hazaribag in the state of Jharkhand in east India, with the ultimate goal of enhancing the development of evidence-based policies to reduce the burden of disease due to MIP in this region of India.

## 2. Methods

### 2.1. Study Sites/Design and Population

**Figure 1:**
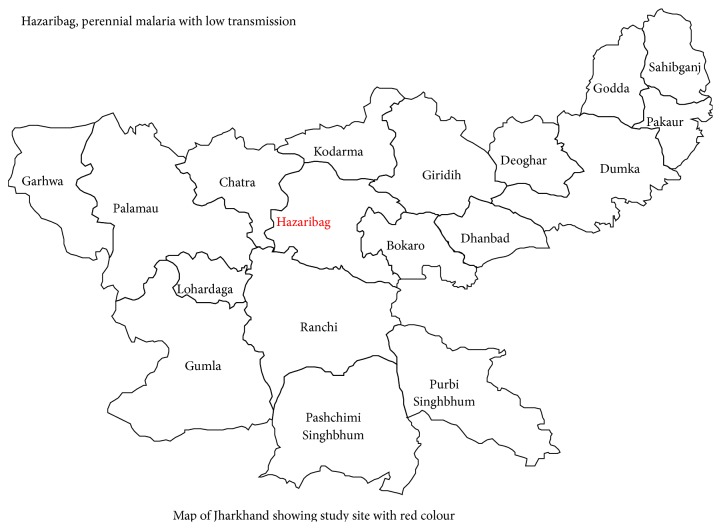
Map of Jharkhand with study site, Hazaribag shown in red.

This study consisted of cross-sectional surveys conducted in three units, that is, antenatal care units (ANCs), delivery units (DUs), or the inpatient antepartum ward of Sadar Hospital in Hazaribag districts of Jharkhand, India (Figure 1). Jharkhand had a yearly average slide positivity rate (SPR) for symptomatic individuals of 6.8% over the last three years with* P. falciparum*,* P. vivax*, and mix infection accounting for 44%, 44%, and 7% of the cases, respectively [28]. The province of Jharkhand in eastern India is one such area where malaria is rampant. The complexity and magnitude of malaria in the central eastern part of India deserve special mention and attention as the central eastern state contributes 15–20% of total malaria cases in the country as per the Draft on National Policy on Tribals by Government of India, 2005. The investigation is conducted in the Jharkhand state emphasizing tribal dominant area (total population according to 2001 census is 31 463 866), and the state of Jharkhand is selected to represent an endemic with stable transmission of malaria, with a total of 230 686 malaria cases reported in 2009, of which 39.53%, 52.64%, and 7.83% were due to* P. falciparum*,* P. vivax*, and mix infection, respectively [29]. The present study was carried out in Hazaribag district, considered to be a malaria endemic area in the state of Jharkhand.

*Hazaribag* (total population according to 2011 census is 1,734,005) is selected to represent a rural-cum semiurban district with low but perennial transmission of malaria. Hazaribag had a yearly average SPR of 7.3% for symptomatic individuals over the last three years, with* P. falciparum*,* P. vivax*, and mix infection accounting for 14%, 73%, and 13% of the cases, respectively [30]. The majority of the indigenous population is mix of tribals, schedule caste, schedule tribes, and other castes, exceptionally typical social stratification having gender disparity. Moreover, the district and state lie in the tropical zone with an annual rainfall of 1234.5 mm with favorable geoclimatic and ecological conditions conducive for perennial malaria transmission. The climatic conduciveness of the investigated district can be best visualized in the self-explanatory Supplementary Figure-1A (see Supplementary Material available online at http://dx.doi.org/10.1155/2015/740512). Most interestingly, with the monthly climatic temperature when compared with monthly malaria episode, we observed significant correlation between ambient temperature and subsequent rise and fall in malaria episode as shown in Supplementary Figure-1B. The recent (2010–2012) data on malaria epidemiology has been analyzed during investigation in this project and we observed the increasing trend of malaria episodes as shown in Supplementary Figure-2A-C, despite consistent interventions and preventive measures implemented by various national and international bodies.

Thus, the selected study district is meant to provide a representation of typical conditions that would be found in malaria endemic districts of Jharkhand.

The District Level Household and Facility Survey conducted between December 2007 and April 2008 revealed that 56% of women had at least one antenatal clinic (ANC) visit and 18% overall had institutional deliveries including 59% in urban areas but only 13% in rural settings [31]. Sadar Hospital, the district hospital for Hazaribag district, serves a predominantly rural population and has a separate obstetric unit with 40 beds, with a high volume of annual deliveries ranging from an average of 4800 to 5500 per year in 2010 to 2013. The Sadar Hospital also has a high volume of ANC visits including an average of 5200 to 6600 per year from 2010 to 2013.

### 2.2. Screening and Enrollment

The study had two components with recruitment targeted to all the women presenting to antenatal care unit (ANC) and delivery units (DUs). For the ANC component, pregnant women aged ≥17 years who reported to the study site for routine care were screened and enrolled; those were willing and consented to participate in our study. For the DU component, women aged ≥18 years who presented for delivery and were willing to provide written informed consent were enrolled. Inclusion in the study protocol was based on the considerations like residency and availability status in the study region, no history of hereditary diseases and/or no known severe disease at the time of conceiving and/or at first ANC attendance, voluntary and consented participation in our study, and no immediate illness due to other infectious diseases or malaria in preconception and/or during present pregnancy at the time of first attendance at ANC. Exclusion from study was based on either refusal to give signed consent or unwilling for sampling, clinically suspected or identified cases of HIV and hepatitis B infection, and stringently those who are apparently and at first sight so weak due to unknown reason compounded by pregnancy that may not sustain sampling stress and may lead to undesired complications.

Detailed strategy of enrollment, sampling procedures, and broad groups were as described; recruitment and enrolment took place from September 2012 to December 2013. Of 1890 pregnant women screened during their ANC visits, 1746 were willing to understand our study protocol, out of which 1715 consented and agreed on peripheral sampling and 31 refused to participate in the study. Thus, we enrolled 1715 subjects, interviewed by trained technical staff, and, upon pregnancy screening report and based on other clinical investigations, divided them into the two broad groups, that is, pregnant and nonpregnant women group consisting of 1271 and 444 subjects, respectively. The nonpregnant group was subdivided into women with malaria and healthy women without malarial complications, consisting of 227 and 217, respectively. In the delivery unit, 870 pregnant women were screened and enrolled as shown in schematic flow chart in Supplementary Figure-3. All the women at each attendance underwent clinical investigations, parasite slide examination, and measurement of auxiliary body temperature before enrollment and we found 68 and 37 MIP cases at ANC and DU, respectively. In ANC, we found 59, 3, and 6 cases of* P. vivax*,* P. falciparum*, and mix infection, respectively, whereas at DU, we found 32, 2, and 3 cases of* P. vivax*,* P. falciparum*, and mix infection, respectively, at Sadar Hospital, Hazaribag (Table 1). The controls for malaria in pregnancy were malaria in women without pregnancy group in addition to healthy women; those are without pregnancy having no known diseases including malaria at the time of sampling.

### 2.3. ANC Procedures

Trained study personnel interviewed the enrolled women and collected information on sociodemographic characteristics (i.e., date of birth, socioeconomic status, and literacy), reproductive history including gravidity, history of fever and antimalarial drug use, and use of antimalarial prevention measures. A complete physical examination including the determination of gestational age was assessed by palpation of uterine fundus height combined with information on last menstrual period; measurement of auxiliary temperature with digital thermometer and other vital signs was also performed. Peripheral venous blood (3–5 mL) was collected from all the attendees for malaria blood film preparation, rapid diagnostic test (RDT), and haemoglobin determination apart from other biochemical and molecular investigations. Women with positive RDT results or who were anaemic were referred immediately to the hospital physician for treatment. The hospital staffs were informed of additional parasitaemic individuals identified through blood smears so that they could be appropriately treated.

### 2.4. DU Procedures

Pregnant women enrolled at the DUs were interviewed, with data collection focused on sociodemographic and anthropometric characteristics, obstetric complications, history of fever and antimalarial use during pregnancy and the use of antimalarial prevention measures, birth outcome, and mode of delivery. Peripheral venous blood (3–5 mL) was collected after delivery for malaria blood film preparation and/or rapid diagnostic test (RDT) and haemoglobin determination apart from other biochemical and molecular investigations. Women with positive RDT or blood smear results were referred for treatment. Apart from malaria prevalence study in DU, we have also collected clinical and demographic data and samples based on the mode of delivery, that is, normal, caesarean, and stillbirth delivery, and further on the mode of birth/delivery outcome, that is, preterm, postterm, and term delivery; details were presented in Supplementary Table-1. To assess the gestational age, we mainly adopted the simplest method, that is, symphysis-pubis fundal height (SFH) measurement (also known as palpation of uterine height measurement), most widely used method over the globe especially in resource poor settings like ours. Assessments were performed by trained nurses followed by gynaecologist. However, in case of any undesired measurement or dought over positioning of foetus, they were confirmed by ultrasound to record the gestational ages.

### 2.5. Laboratory Procedures

Thick and thin smears prepared from peripheral blood of ANC and DU subjects were Giemsa-stained and examined under high power. The parasite density was evaluated by counting the number of asexual forms of parasites for every 200 leukocytes, assuming a leukocytes count of 8000 leukocytes/*μ*L of blood [32]. The thin film was used to identify the* Plasmodium* species. All slides were cross-checked using stringent diagnostic criteria to diagnose* Plasmodium* infection with our trained technical staff. The commercial (RDT kit) First Response Malaria pLDH/HR2 combo test kits (Premier Medical Corporation, Mumbai, India) were also used as per the manufacturer's guideline as a screening tool for diagnosing malaria in pregnant women. We have used the PCR technique also to diagnose malaria but in selective samples not in all the samples due to budgetary constraint. The selective samples were all the MIP positive samples at ANC and DU verified by PCR, those subjects who were disputed on microscopy and RDT also verified by PCR, and clinically most suspected cases with strong sign and symptoms but microscopically negative samples were also verified by PCR.

### 2.6. Haemoglobin Concentration

Haemoglobin (Hb) levels were recorded at the first ANC and DU visit. Determining the concentrations of haemoglobin (Hb) was performed in peripheral blood samples using a portable HemoCue haemoglobinometer (HemoCue AB, Ängelholm, Sweden) as stated by the manufacturer. The concentration of Hb was recorded on the study questionnaire and double-checked by the laboratory technician. Women were classified as anaemic (Hb < 11 g/dL) and then categorized as being moderately to severely anaemic, with haemoglobin <8 g/dL and <7 g/dL, respectively, as the primary outcome, and being mild to nonanaemic (Hb ≥ 9 g/dL) according to [33, 34].

### 2.7. Study Definitions

Severe malaria was defined as a malaria attack associated with any of the following: cerebral malaria, severe anaemia, renal failure, pulmonary oedema, hypoglycaemia, shock, spontaneous bleeding, or repeated convulsions [35]. Maternal height and weight were taken at the first visit to ANC and DU; based on this information, the body mass index (BMI) was calculated as weight (kg) divided by the squared height (meters); a low BMI was defined as a BMI < 22.0 kg/m^2^. A documented fever was defined as an auxiliary temperature ≥37.5°C.

### 2.8. Ethics Statement and Subject Consent

All human blood samples used in this study were collected after obtaining written consent from the study participants under protocols activities approved by the Institutional Ethics Committee (IEC) of the Vinoba Bhave University, Hazaribag, Jharkhand, and human ethical guidelines as reflected in the guidelines of the Medical Ethics Committee, Ministry of Health, Government of India. Present study does not involve any minor/children. Thus, signed and written approval was given by adult subject herself. All study participants were included only after informed consent. The study protocol and consent proposal are approved from IEC, VBU, having memo number VBU/R/888/2012, dated 05-06-2012.

### 2.9. Data Management and Analysis

All clinical, demographic, and anthropometric information were carefully checked for correctness and inconsistencies were resolved before analysis. Data were entered in MS-Excel and analyses were performed using SPSS version 16 (SPSS Inc., Chicago, IL, USA) and Graphpad Prism version 5.0 (GraphPad Software, Inc., CA, USA). For comparisons of means between two groups of subjects, Student's *t*-test was used for evaluating significance for normally distributed data and when data were not normally distributed; nonparametric tests (Mann-Whitney *U*) test were used to analyze the data. Categorical data are presented as frequency counts (percent) and compared using the Chi-square or Fisher's exact statistic as appropriate. Continuous data are presented as means (± standard error) and compared using the *t*-test or analysis of variance as appropriate. The age of the recruited subject was between 18 and 37 years, whereas mean age was 26.7 years. We have presented participants' ages in ranges based on their responses (Supplementary Table-1). Risk factors for either* P. falciparum* or* P. vivax* parasitemia were evaluated by univariate analysis and then adjusted for significant predictors in multivariate analysis. Simple and multiple logistic regressions were used to analyze potential risk factors associated. Precisely, to investigate the association between the various independent variables (selecting only strong epidemiological and biological plausibility for association) and malaria parasitemia, we began by performing simple logistic regressions with each independent variable. Next, we applied multiple backward logistic regression models and all covariables present in univariate were kept in model, independent of their significance, in univariate analysis due to their possible relevance in the final results; thus, we could analyze their possible influence when considered together with the other variables. Similar strategies were followed for factors associated with haemoglobin and anaemia during pregnancy and malaria in pregnancy; risks were assessed using haemoglobin or anaemia as dependent variables and all other factors as independent variables. The differences were considered statistically significant when the *p* value obtained was <0.05.

## 3. Results

### 3.1. Antenatal Clinics

Most pregnant women attending ANC were in the 18 to 38 years of age range and had some level of formal education (Supplementary Table-1). The vast majority of participants were Hindi speaking (97.6%) and nonsmoking (98.7%). Most owned their own home (75.4%) and were engaged in household work (76.7%) with a small proportion involved in farming (12.3%). They had attended a median of one ANC visit (range 0–9) during their current pregnancy and almost one-third of the attendees were primigravidae (33.3%). Slightly more than half of participants presented to the ANC in the latter half of pregnancy whereas 44.6% presented prior to 20 weeks. Less than half of the participants reported taking iron/folate supplements (46.3%) while 33.2% were taking multivitamins. In terms of malaria prevention activities, most pregnant women reported having untreated bed nets in their homes and using them recently, but very few had ITNs (Supplementary Table-2). Similarly, only 9 of the women were taking prophylaxis for malaria and most of them (7/9, 78%) were unable to identify the drug they were taking and the rest (two), who were able to identify the drug, were taking chloroquine.

A positive diagnostic test for malaria was obtained in 5.4% (68/1271) of the total cohort (Table 1). Blood smears for malaria were positive in 4.3% of pregnant women while an additional 14 (1.1%) women had positive RDTs. The mean density of parasitemia in the 54 women with positive blood smears was 63,236 asexual forms/*μ*L (range 600–489,000).* P. falciparum* was identified in 4.4% of parasitaemic individuals while* P. vivax* was found in 86.8% and 8.8% of infections were mixed. Peripheral parasitemia was over four times more likely among women living in rural areas when compared with those from urban or semiurban subjects (OR 4.36, 95% CI 2.48–7.32) and among primigravidae and secundigravidae relative to multigravidae (OR 4.23, 95% CI 2.15–8.82). Parasitaemia was more commonly encountered in pregnant women who had a history of fever within the week prior to enrollment or were febrile at the time of the study visit (4.2% versus 2.3%, *p* = 0.02). The majority of positive malaria tests occurred from July to January with the greatest number in between August and October, corresponding to the monsoon season. Further multivariate analysis was performed in order to identify the association between specific demographic, socioeconomic, and malaria prevention activities and the risk of parasitemia. Among pregnant women attending ANCs, first/second pregnancies, fever in the past week, and residence in rural areas were significantly associated with peripheral parasitemia as shown in Table 2.

### 3.2. Delivery Units

Like the ANC cohort, most pregnant women attending DUs were aged 20–36 years and had some level of formal education (Supplementary Table-1). All were nonsmokers (100%) and nearly all spoke Hindi (97.2%). Most owned their own home (73.9%) and were involved in household work (84.3%); a minority engaged in farming (14.6%). Study participants had attended a median of three ANC visits (range 0–9) and about slightly less than two-thirds were primigravidae and secundigravidae (Supplementary Table-1). The majority of pregnant women reported having untreated bed nets in their homes and using them recently but ITN ownership was uncommon (Supplementary Table-2). Only three women were taking chemoprophylaxis for malaria and none knew the name of the medication that they were taking. Only 4.3% of the women enrolled at the DUs had peripheral parasitemia (either a positive blood smear or RDT).* P. falciparum* was identified in 5.4% (2/37),* P. vivax* in 86.5% (32/37), and mixed infection in 8.1% (3/37). The mean density of parasitemia in the women with positive blood smears was 16,395 asexual forms/*μ*L (range 870–65,000). The peripheral parasitemia density was significantly higher in primigravid women than in those who had one or more prior pregnancies (mean ± SD of 36, 600 ± 9, 743 versus 7, 532 ± 4623 asexual forms/*μ*L, resp.; *p* = 0.002). Pregnant women with peripheral parasitemia were more likely to have either a self-reported fever or fever measured at enrollment than those who were aparasitaemic (36.4% versus 9.2%, *p* = 0.005). A sizable proportion of women presenting to the rural origin were parasitaemic as compared to semiurban and urban origin and this difference was significant (OR 4.36, 95% CI 2.48–7.32, and *p* = 0.0001) (Table 2). Primigravidae and secundigravidae also were more likely to be parasitaemic, and difference was significant (OR 4.23, 95% CI 2.15–8.42, and *p* = 0.0001). Asymptomatic malaria infections were present in 70% of women with peripheral parasitemia (26/37) as compared to 30% symptomatic infection (11/37). Pregnant women with peripheral parasitemia were more likely to have either a self-reported fever or fever measured at enrollment than those who were aparasitaemic (28.3% versus 9.2%, *p* = 0.004).

As observed in the ANC participants, most episodes of parasitemia occurred in July to September during the monsoon season. For DU participants with peripheral parasitemia, 83.7% had anaemia as compared to 47.6% of those who did not have parasitemia (*p* = 0.004). More women with peripheral parasitemia had severe anaemia (5.7%) than those without parasitemia (2.6%) and the difference was significant (*p* = 0.02).

Multivariate analysis revealed a significant association between peripheral parasitemia and primigravidae and secundigravidae, fever within the last week, and semiurban and rural residency status as shown in Table 2.

### 3.3. Association between Pregnancy and Asymptomatic* P. vivax* with Haemoglobin

**Figure 2:**
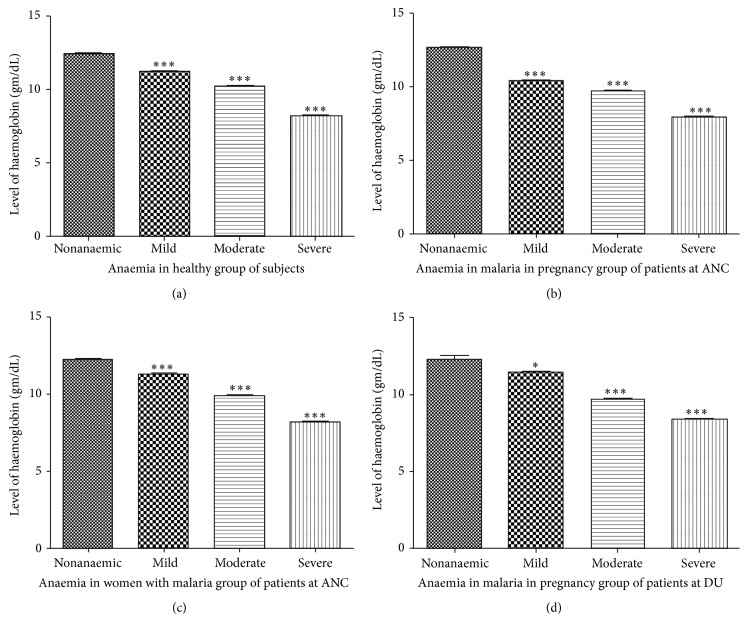
Level of haemoglobin as classified anaemia in malaria infected subjects screened at antenatal care (ANC) unit and delivery unit (DU) in stratified group as (a) anaemia in healthy group of subjects, (b) malaria in pregnancy group of patients among ANC attendees, (c) women with malaria group of patients without pregnancy, and (d) malaria in pregnancy group of patients among DU attendees. Data is presented as mean and error bar represents the plus or minus SE ^*∗*^ *p* ≤ 0.01, ^*∗∗*^ *p* ≤ 0.001, and ^*∗∗∗*^ *p* ≤ 0.0001 compared with nonanaemic women using paired *t*-test through Graphpad Prism version 5.0.

Anemia is the most prominent hematological manifestation of malaria infection. Hemoglobin concentration is the best characterized method and well accepted indicator for diagnosis of anemia and assessment of severity. In addition to this, it is regarded as one of the most serious global public health problems which prompted us to investigate the status of hemoglobin and severity of anemia in Jharkhand population, as anaemia is particularly high for women with no education (74%), women from the scheduled tribes (85%), and women in the two lowest wealth quintiles (over 70%). The prevalence of anaemia among adults in Jharkhand is higher than in almost all other states in India (national family health survey, NFHS-3 India, 2006). Anaemia was prevalent among ANC participants whereas severe anaemia was reasonably observed in the investigated cases (Supplementary Table-1). More than two-thirds of the DU participants were anaemic whereas 7.8% had severe anaemia (Table 1). Of these ANC and DU participants, the prevalence of mild, moderate, and severe anaemia is shown in Figures 2(a)–2(d).

### 3.4. Association of Asymptomatic Infection with Malaria during Pregnancy at ANC and DU Subjects

Clinical malaria cases are suspected and investigated on the basis of malaria associated sign and symptoms in various studies including community based epidemiological studies; and based on the prevalence of sign and symptoms, we interestingly observed in our study that 70.6% (48/68) of the positive cases of malaria in pregnancy subjects at ANC were asymptomatic with peripheral parasitemia compared to 29.4% symptomatic MIP cases, whereas 75.7% were asymptomatic cases with peripheral parasitemia compared to 24.3% symptomatic infection during malaria in pregnancy at DU. Based on the data collected on sign and symptoms from the pregnant women attendees at ANC and DU subjects, we performed positive predictive value (PPV) (Table 3) and multivariate (Table 4) analysis to further consolidate our observation and to explore the association between symptoms and malaria infection during pregnancy. For positive predictive value (PPV), fever, history of fever, body pain, headache, dizziness, vomiting, and convulsions were evaluated at ANC and DU as shown in Table 3. Almost all the predictive values for respective symptoms were observed to be very much low except for history of fever, which is relatively higher than the others only, despite being the highest among all at both ANC and DU. However, the positive predictive value for history of fever at DU was slightly higher than ANC. None of the predictive value for any sign and symptoms was neither nearly 50% or even above (Table 3). The prevalence of observed values (%) and frequencies (*N*) for all the signs and symptoms were also presented in Table 3. Further, in applying multivariate model, we analysed any symptoms, fever, history of fever, headache, dizziness, and vomiting at ANC and DU as shown in Table 4. We observed that presence of any symptoms, history of fever, headache, dizziness, and vomiting were not significantly associated with incidence of malaria during pregnancy at ANC, whereas only fever was found to be significantly associated at ANC as shown in Table 4. However, in case of DU subjects, all the symptoms were not significantly associated except fever and history of fever which were significantly associated with incidence of malaria as shown in Table 4. Thus, based on the observation and analysis, we can infer that majority of the sign and symptoms have not been shown or trended to be significantly associated, except fever and/or history of fever that have some degree of significant association with malaria in pregnancy at ANA and DU in multivariate analysis. The absence of higher percentage of positive predictive value for all the symptoms as well as lower prevalence of observed value and frequency can also be regarded as an indicative of nonassociation of sign and symptoms with incidence of malaria during pregnancy at both ANC and DU. As majority of subjects were infected with* vivax* strain as described earlier (Table 1) both at ANC and DU, thus, view of nonassociation of sign and symptoms with the incidence of malaria during pregnancy can be coined and corroborated with asymptomatic* Plasmodium vivax* infection in the present study both at ANC and DU.

### 3.5. Risk Factors Associated with Anaemia in Overall Study Cohort and Malaria in Pregnancy at ANC and DU Subjects

Multivariate logistic regression showed that malaria infection, ferritin, iron, haemoglobin, and formal education were significantly associated with a higher risk of anaemia in overall cohort (*N* = 1271 at ANC and *N* = 870 at DU) as well as in malaria in pregnancy at ANC (*N* = 68) and DU (*N* = 37) subjects as presented in Table 5. The highest (adjusted odds ratio in multivariate analysis) risk factor associated with anaemia was observed with haemoglobin level, followed by presence of malaria infection in both malaria in pregnancy and overall study cohort at ANC and DU as shown in Table 5. However, ANC subjects have shown relatively higher risk ratio association of anaemia with haemoglobin and malaria compared to DU subjects (Table 5). Comprehensive results of univariate and multivariate analysis are shown in Table 5.

## 4. Discussion

The estimate of malaria in pregnancy continues to be grave concern for community reproductive health care management across the tropical region including India, up to the level of pacifying the concept of healthy mother and healthy baby of National Family Welfare Programme. In fact, situation is much more aggravated in developing countries like India, where poverty, illiteracy, geographical diversity, socioeconomic disparities, and multiple pregnancies take their toll of mother's health.

Among the prominent findings of the present study, we found 5.4% and 4.3% malaria during pregnancy at ANC and DU, respectively, as compared to only 1.8% and 1.7% at ANC and DU, respectively, reported by Hamer et al. [23] from the series of cross-sectional and multicentric study in Jharkhand. However, our study design is slightly broader than the earlier investigation from Hamer et al. [23] in terms of subject stratification, as we have also taken into account women with malaria without pregnancy, and the prevalence of malaria was found to be 13.2%, which itself reflects the importance of the investigated region and population under malaria sensitive zone. However, our study lacks the difference of investigating placental malaria. The pondering difference in the prevalence of malaria during pregnancy between our investigations, though we have selected only one centre in one district, that is, Hazaribag, Jharkhand, as compared to three centres from two districts, that is, Ranchi and Gumla of Jharkhand by Hamer et al. [23], may be attributed to various other reasons but primarily linked to the selection of study sites. As Ranchi is an urbanized capital with lots of high-tech development in and around the city, local and buffering populations are much more educated, aware of practicing healthy life style and various diseases prevention strategies including malaria, having high socioeconomic status, excellent with a choice of health facility compared to the rest of the districts of Jharkhand state, and most importantly less malarious than almost 20 other districts of Jharkhand as far as malarial epidemiology is concerned in last ten years [29]. Thus, selected site by Hamer et al. [23] may not be the true representation of the malaria scenario and rather burden of malaria during pregnancy in Jharkhand but absolutely true as far as the outcome of the project is concerned. However, our results of higher prevalence of malaria in pregnancy are in accordance with the earlier observations (ranging from 1.7% to 20%) across India [21, 23, 36, 37]. Most of these studies focused on pregnant women with selective approach, tend towards screening for mostly febrile, or had a recent history of fever cases and thus may have had a selection bias towards expecting higher malaria rates. This approach, targeting malaria diagnostic and treatment for symptomatic pregnant women, is consistent with India's National Vector Borne Disease Control Programme guidelines [38]. In contrast, all pregnant women were evaluated in the current study regardless of classical symptoms and, interestingly, we observed well that over 70% of the pregnant women in ANC and DU had asymptomatic malaria during pregnancy, which suggests the region specific intervention. The broader spectrum of screening strategies was in accordance with earlier investigation in this region [23], though our observations are notably varied from their observations as far as asymptomatic malaria during pregnancy is concerned. Infection from* P. vivax* in pregnancy has conventionally been regarded less severe as compared to* P. falciparum* malaria. Interestingly, we reported that majority were infected with* P. vivax* infection during malaria in pregnancy. This observation may be attributed to the lack of placental sequestration in* P. vivax* infection and the parasite tropism for reticulocytes accounting for a milder form of anaemia [39, 40].

The higher prevalence of malaria in women without pregnancy and with pregnancy, irrespective of ANC and DU attendees' location of residence, that is, rural, urban, and semiurban, suggests that Hazaribag and its buffering zone have perennial rate of malaria transmission. Therefore, populations of all age groups including pregnant women are at potential risk of getting malaria infection even irrespective of transmission season, though peak was observed in postmonsoon season. Apart from this, there is significant lack of education, general awareness towards health issues, congenial environmental factors for vector growth and survival, and most importantly sizable population lack access to vector control methods or limited access to antimalarial drugs. People residing below poverty line linking to malnutrition and anaemia may be plausible reasons for various opportunistic infectious diseases including malaria.

Interestingly, insecticide residual spray (IRS) of home, which is usually conducted by government agencies, was reported more in rural areas as compared to urban and semiurban zone of Hazaribag, though its seasonal usage of IRS in those areas regarded as perennial transmission may be suggestive of vector resistance and subsequent higher prevalence of disease. Our observations warrant the potential need to enhance the IRS and distribution of ITNs in and around the investigated district.

We report that* P. vivax* is associated with a high burden of anaemia and remarkable severe anaemia during pregnancy and malaria in pregnancy in endemic population of Hazaribag.

Overall, there was significant burden of anaemia among women in Jharkhand and particularly during pregnancy [23]. Our observations regarding anaemia are in accordance with the findings from other studies in Jharkhand [23], across India [41, 42], and most relevant study by Nosten et al. [43] in which they have demonstrated that women who had malaria at any time were more likely to be anaemic than women without malaria. Among multifactorial involvement in malarial anaemia are included haemolysis of parasitized erythrocytes and increased clearance of nonparasitized ones as well as an inadequate bone marrow response [44]. It has been suggested that pregnancy has also confounding association with anaemia and malaria [43, 45] and* P. vivax* has shown 2-fold higher risk of moderate anaemia than uninfected subject [46, 47].

Thus, regardless of transmission level and the level of prepregnancy immunity against malaria, maternal anaemia remains the most frequent adverse consequences of malaria during pregnancy [48]. The symptoms and complications of malaria in pregnancy vary according to malaria transmission intensity in the given geographical area and the individual's level of acquired immunity. In low-transmission settings, where women of reproductive age have relatively little acquired immunity to malaria, MIP is associated with anaemia, an increased risk of severe malaria. This may lead to spontaneous abortion, stillbirth, prematurity, and low birth weight [49, 50]. In such settings, malaria affects all pregnant women, regardless of the number of times they have been pregnant. In pregnant women, additional sequestration of malaria infected erythrocytes occurs in the placenta. Pregnant women therefore suffer disproportionately from severe anaemia as a result of infection [14]. Our observation is also substantiated by the fact that the majority of malaria infections in pregnancy remain asymptomatic or paucisymptomatic and yet are a major cause of severe maternal anaemia and low birth weight, especially in the first and second pregnancies [22, 23]. In areas with stable but low transmission like our investigated area and certainly in areas with unstable and exceptionally low transmission, infections can become severe in all gravidae groups because most women of childbearing age in these regions have low levels of prepregnancy and pregnancy-specific protective immunity to malaria [14].

High prevalence of anaemia was observed and strongly correlated with asymptomatic* P. vivax* infection. This prevalence is similar to that reported by Brutus et al. [47] and Douglas et al. [46, 51]. Recent work has shown that, in Papua New Guinea and Papua, Indonesia, mixed infection causes more severe haematological impairment than infection with either species alone [52–54]. The impact of* Plasmodium vivax* infection on haemoglobin concentration varies from negligible to dramatic [52, 55–57]. The clinical consequences of the reduction in haemoglobin depend on the haemoglobin concentration prior to infection. Although the spectrum of anaemia seen with* vivax* infection is reasonably well documented, the clinical, developmental, and socioeconomic consequences are largely unknown. Population-based estimates of mortality in severely anaemic individuals with* vivax* malaria have not been established but recent studies from Latin America, New Guinea, and the Indian subcontinent have identified deaths in patients with severe* vivax* anaemia [52, 55, 58, 59]. However, authors did not establish the extent to which anaemia contributed to those deaths.

The very low rate of ownership of insecticide treated bed nets (ITNs) and awareness suggests that this component of the enhanced malaria control programme (EMCP) has not effectively reached this vulnerable population although it was encouraging to find that many households had bed nets and that they were used on a regular basis. However, our investigation suggests that approaches for ITN distribution and enhancing community awareness about the importance of their use need to be addressed as similarly observed and proposed by earlier investigation in adjacent region by Hamer et al. [23].

Despite the change in drug policy in 2008 in the studied state (Jharkhand), the availability and implementation of combination therapy, that is, artesunate plus sulfadoxine pyrimethamine, are a major concern. It has been well documented that chloroquine resistance has been rising in India [60–63]; this drug was recommended for malaria prophylaxis in pregnant women in high risk areas as reported by Hamer et al. [23], though it has been discontinued since recommendation. Presently, quinine sulphate was recommended for malaria prophylaxis in pregnant women in the investigated area irrespective of gestational age. However, this is partly in accordance with The Directorate of National Vector Borne Disease Control Programme (NVBDCP) and current WHO guidelines suggesting prophylaxis for trimester based treatment of malaria during pregnancy as quinine for first trimester and subsequently ACTs in the second and third trimester of pregnancy (http://www.nvbdcp.gov.in/Doc/Diagnosis-Treatment-Malaria-2013.pdf). Since the intensity of transmission and the prevalence of malaria in pregnant women in Jharkhand are comparatively lesser than in many areas in sub-Saharan Africa, notably, sulfadoxine pyrimethamine was commonly used in Africa as intermittent preventive treatment of pregnant women (IPTp) [15], which may not be presently suggestive priority for Jharkhand to implement IPTp though it may be considered as an alternative to the priority failure strategy. The top priority for Jharkhand should be on preventive measures like improved availability, awareness and uses of ITNs by pregnant women, and well organised IRS system. In addition, we recommend much more stringent and frequent screening and diagnosis using conventional and RDTs irrespective of classical malaria symptoms to pregnant women in all the trimesters. Most importantly, in view of sizable prevalence based on hospital study and potential risk for population at large in the investigated region, we are also suggestive of dedicated active and passive surveillance for MIP at the community level like regular malaria surveillance under India's NVBDCP. This strategy alone could potentially reduce the burden of MIP while limiting the potential for antimalarial resistance to develop due to the widespread use of drugs for chemoprophylaxis. The present study shows two important findings; that is, the observed predominant prevalence of asymptomatic infections differs from that of symptomatic disease and marked alteration in haematological indices during* P. vivax* infection with pregnancy synergistically contributes to maternal anaemia in a low and perennial malaria transmission setting.

One major limitation of this study is that we were unable to access the placental malaria due to limitation of our study design. Although the study was restricted to women delivering in the hospital, a sizable number of (more than 60%) women give birth outside Sadar Hospital, Hazaribag.

Further, a longitudinal study instead of cross-sectional one would have provided better estimate of MIP in this region and probably our study design may have given underestimate as compared to actual risk population. This has also been apprehended and suggested by Hamer et al. [23]. Despite these limitations, this study provides important data on the epidemiology and clinical implications of* vivax* malaria during pregnancy and delivering at Hazaribag district Sadar Hospital. In spite of restricted and facility based study, we preferentially covered marginalized, tribes, and remote population of the investigated rural-cum semiurban district, Hazaribag. The majority of the districts and particularly malaria endemic districts in Jharkhand have similar geographical, socioeconomic, demographic, literacy, and basic amenities including health facility and awareness. Thus, our observation may be utilized for baseline information for further comprehensive and multicentric study design, in strengthening MIP associated preventive measures and screening methods within the state of Jharkhand.

## 5. Conclusion

As the global control and elimination of malaria progress,* P. vivax* is set to become the dominant* Plasmodium* species [64]; yet, the health, developmental, and socioeconomic consequences of* vivax* malaria and* vivax*-associated anaemia have received very little attention. Salient findings of this study are as follows:

- There is high prevalence of anaemia during pregnancy and in delivering women in the malaria endemic population of Hazaribag, Jharkhand.
- Prevalence of anaemia is significantly associated with* Plasmodium vivax* infection during pregnancy and in delivering women.
- The most significant observation was the high prevalence of asymptomatic* P. vivax* infection at both ANC and DU.

Taken together, these observations are quite indicative and emphasize the need to actively diagnose and treat malaria infection during ANC visit in the areas of perennial transmission. Additionally, in view of the sizable population at risk in this malaria endemic region of India, we are suggestive of few priority practice amendments and reorientation of policies for MIP prevention strategies:

- There is an urgent need to enhance the ITN availability, use, and awareness both in population and health worker.
- Distribution of ITNs at first ANC visit will be lucrative alternative for preventive strategy.
- There should be priority consideration of early case detection and management of asymptomatic pregnant women through restructuring the need of active and passive surveillance strategy in endemic as well as in nonendemic zone.
- In view of the asymptomatic prevalence of coinfection, we need to further strengthen and emphasize the robust screening strategies, curative attention, and safe treatment facilities at the community level health centres.

Further, integrated investigation is desperately needed to understand the magnitude and prevalence of asymptomatic malaria infection linking as an important infected reservoir to continue malaria transmission. Precisely, our finding highlights the public health importance of integrated genus-wide malaria control strategies using diagnostic tests including RDTs and ensuring the availability of safe and effective drugs for the treatment of pregnant women in areas of* Plasmodium* coendemicity.

## Supplementary Material

Extensive malarial epidemiology related to study sites mentioned in methodology section, detailed demographic information about the subjects investigated during this study as part of method section of manuscript text and some additional and supportive findings mentioned in the results section, which further consolidate and substantiate our observation of asymptomatic prevalence of malaria anaemia in the investigated region has been given in Supplementary material attached to this article.

## Figures and Tables

**Table 1:** Parasitaemia, reported fever, and anaemia among pregnant women attending antenatal clinics and delivery units.

	Antenatal clinics	Delivery units
	*n* = 1271	*n* = 870
	*N* (%)	*N* (%)
Peripheral parasitaemia		
Overall	68 (5.4)	37 (4.3)
Falciparum	3 (0.23)	2 (0.22)
Vivax	59 (4.6)	32 (3.67)
Mixed	6 (0.47)	3 (0.34)
By gravidity		
Primigravid	21/423 (4.9)	11/338 (3.2)
Secundigravid	38/578 (6.6)	15/209 (7.1)
Multigravid	9/270 (3.3)	11/323 (3.4)
Report of fever within 1 week	167 (13.1)	93 (10.6)
Anaemia	1093 (86)	626 (72)
Severe anaemia	148/1093 (13.6)	49/626 (7.8)

**Table 2:** Factors associated with peripheral parasitemia during malaria in pregnancy using univariate and multivariate analysis.

	Peripheral parasitemia % (Positive/total)	Adjusted OR (95% CI)	*p*	Adjusted OR (95% CI)	*p*
Factors at ANC					
1st/2nd pregnancies	6.3 (64/1001)	4.45 (2.32–9.61)	0.0001	4.23 (2.15–8.42)	0.0001
3rd or greater pregnancies	1.4 (4/270)	1		1	
Age < 20	7.2 (12/166)	1.43 (0.34–3.76)	0.052	1.31 (0.26–2.84)	0.076
Age ≥ 20	5.0 (56/1105)	1		1	
Fever within past week	16.1 (27/167)	4.42 (3.64–8.21)	0.002	4.62 (3.73–9.83)	0.001
No fever within past week	3.7 (41/1104)	1		1	
Bed net use^*∗*^	7.6 (42/563)	1.12 (0.27–2.47)	0.072	1.37 (0.48–3.24)	0.084
No bed net use	6.7 (26/374)	1		1	
Rural	7.1 (61/857)	4.21 (1.53–5.21)	0.003	4.36 (2.48–7.32)	0.0001
Not rural	1.7 (7/414)	1		1	
Tribal caste	6.3 (23/363)	1.26 (0.64–2.96)	0.054	1.42 (0.81–3.75)	0.12
No tribal caste	4.9 (45/908)	1		1	
No formal education	6.1 (22/357)	1.22 (0.42–2.46)	0.065	1.34 (0.68–3.92)	0.084
Formal education	5.0 (46/914)	1		1	
Factors at DU					
1st/2nd pregnancies	5.8 (32/547)	3.9 (0.97–11.56)	0.004	3.62 (0.94–7.83)	0.001
3rd or greater pregnancies	1.5 (5/323)	1		1	
Age < 20	8.2 (28/109)	2.32 (1.32–9.37)	0.062	2.47 (1.17–10.63)	0.14
Age ≥ 20	3.6 (9/761)	1		1	
Fever within past week	13.9 (13/93)	4.47 (1.25–12.42)	0.0001	4.43 (1.38–11.57)	0.0001
No fever within past week	3.1 (24/777)	1		1	
Bed net use^*∗*^	5.3 (27/503)	1.97 (0.83–7.62)	0.084	1.62 (0.58–6.39)	0.27
No bed net use	2.7 (10/367)	1		1	
Rural	7.1 (29/405)	4.22 (0.41–4.51)	0.003	3.87 (0.78–13.62)	0.0001
Not rural	1.7 (8/465)	1		1	
Tribal caste	5.5 (14/251)	1.51 (0.56–3.92)	0.053	1.74 (0.83–5.38)	0.59
No tribal caste	3.7 (23/619)	1		1	
No formal education	5.3 (17/321)	1.46 (1.23–3.17)	0.57	1.62 (0.87–4.63)	0.21
Formal education	3.6 (20/549)	1		1	

^*∗*^ITN use was not evaluated in this model since ITN were very rarely used and because of quite lesser awareness about ITN among women.

**Table 3:** Positive predictive value (PPV) of clinical signs and symptoms for *Plasmodium vivax* infection.

	*N*	Observed value (OV) (%)	Positive predictive value (PPV) (%)	95% CI in proportion of PPV (%)
Sign/symptoms at ANC				
Fever	54	4.2	26	23.5–28.4
History of fever	167	13.1	45	42.2–47.7
Headache	114	8.9	32	29.4–34.5
Body pain	15	1.2	18	15.8–20.1
Dizziness	29	2.3	21	18.7–23.2
Vomiting	22	1.7	23	20.3–25.3
Convulsions	13	1.1	12	10.2–13.7
Sign/symptoms at DU				
Fever	43	4.9	36	32.8–39.1
History of fever	93	10.6	47	43.6–50.3
Headache	172	19.7	33	29.8–36.1
Body pain	23	2.6	26	23.1–28.9
Dizziness	19	2.2	19	16.3–21.6
Vomiting	31	3.5	27	24.1–29.9
Convulsions	14	1.6	21	18.2–23.7

**Table 4:** Association between signs/symptoms and malaria infection using multivariate analysis.

		*n*/*N* (%)	OR (95% CI)	*p* value
Sign/symptoms at ANC				
Any symptoms	No	937/1203 (77.8)	1	0.14
	Yes	20/68 (29.4)	1.3 (0.9–1.9)	
Fever	No	1138/1203 (94.5)	1	0.0003
	Yes	11/68 (16.1)	2.9 (1.6–5.4)	
History of fever	No	1031/1203 (85.7)	1	0.14
	Yes	14/68 (20.5)	1.4 (0.8–2.3)	
Headache	No	1076/1203 (89.4)	1	0.13
	Yes	11/68 (16.1)	1.5 (0.8–2.6)	
Dizziness	No	1168/1203 (97.1)	1	0.16
	Yes	4/68 (10.1)	1.9 (0.7–5.5)	
Vomiting	No	1178/1203 (98)	1	0.21
	Yes	3/68 (4.4)	2.1 (0.6–6.8)	
Sign/symptoms at DU				
Any symptoms	No	623/833 (74.7)	1	0.9
	Yes	9/37 (24.3)	0.9 (0.5–1.7)	
Fever	No	784/833 (94.1)	1	0.01
	Yes	5/37 (13.5)	2.7 (1.2–6.1)	
History of fever	No	770/833 (98.6)	1	0.01
	Yes	7/37 (18.9)	2.5 (1.2–5.1)	
Headache	No	655/833 (98.6)	1	0.46
	Yes	6/37 (16.2)	0.7 (0.3–1.5)	
Dizziness	No	792/833 (95.1)	1	0.12
	Yes	4/37 (10.8)	2.1 (0.8–5.6)	
Vomiting	No	775/833 (93.1)	1	0.12
	Yes	5/37 (13.5)	1.9 (0.8–4.5)	

*n* = observed, *N* = total considered subjects, and OR= odds ratio.

**Table 5:** Risk factors for anaemia in pregnant women (PW) and malaria during pregnancy (MIP) using univariate and multivariate analysis.

	PW	PW	PW	PW	PW	MIP	MIP	MIP	MIP	MIP
	Number	Crude OR (95% CI)	*p*	Adjusted OR (95% CI)	*p*	Number	Crude OR (95% CI)	*p*	Adjusted OR (95% CI)	*p*
Factors at ANC										
Malaria^*∗∗*^										
No	1203	1	0.0001	1	0.0002	9^*∗*^	1	0.0001	1	0.0001
Yes	68	2.7 (1.4–3.8)		2.8 (1.2–3.4)		59^¥^	3.1 (1.7–5.3)		3.4 (1.9–6.5)	
Ferritin (ng/mL)										
<50	117	1	0.002	1	0.001	5	1	0.0001	1	0.0004
≥50	1154	1.8 (1.3–2.3)		2.2 (1.6–3.5)		63	2.1 (1.6–3.3)		2.4 (1.7–4.1)	
Iron (*µ*g/dL)										
≥40	236	1	0.006	1	0.003	10	1	0.001	1	0.0001
<40	1035	1.7 (1.6–2.7)		1.9 (1.8–3.4)		58	2.2 (1.5–3.3)		2.3 (1.2–2.7)	
Haemoglobin (Hb) (g/dL)										
Hb > 11	217	1	0.0001	1	0.0003	19	1	0.0001	1	0.0002
Hb < 11	1054	3.8 (2.3–8.7)		4.2 (2.1–8.8)	0.0002	49	4.8 (1.7–8.1)		5.4 (1.6–8.6)	
Education										
NFE	357	1	0.003	1	0.001	13	1	0.0001	1	0.0001
FE	914	2.1 (1.4–2.9)		2.3 (1.7–3.9)		55	2.2 (1.6–3.5)		2.6 (1.3–3.4)	
Factors at DU										
Malaria^*∗∗*^										
No	833	1	0.0001	1	0.0002	5^*∗*^	1	0.001	1	0.0001
Yes	37	2.1 (1.2–2.5)		2.4 (1.6–3.8)		32^¥^	2.2 (1.4–3.1)		2.8 (1.7–4.7)	
Ferritin (ng/mL)										
<50	205	1	0.004	1	0.0003	7	1	0.0001	1	0.0002
≥50	665	1.4 (1.2–1.7)		1.7 (1.4–2.3)		30	2.1 (1.8–3.8)		2.3 (1.5–3.5)	
Iron (*µ*g/dL)										
<40	683	1	0.002	1	0.0002	31	1	0.006	1	0.0003
≥40	187	1.9 (1.6–3.1)		2.2 (1.3–2.9)		6	2.3 (1.5–3.5)		2.5 (1.3–3.3)	
Haemoglobin (Hb) (g/dL)										
Hb > 11	173	1	0.0001	1	0.0001	8	1	0.001	1	0.0001
Hb < 11	697	3.1 (1.8–5.6)		3.6 (2.1–7.6)		29	4.2 (1.9–7.8)		4.8 (1.6–7.7)	
Education^#^										
NFE	321	1	0.004	1	0.0002	11	1	0.002	1	0.0001
FE	549	1.6 (1.1–1.8)		1.8 (1.4–2.6)		26	1.7 (1.5–2.6)		2.1 (1.7–3.6)	

PW = pregnant women, MIP = malaria in pregnancy, ANC = antenatal care unit, and DU = delivery unit; ^#^NFE = no formal education, FE = formal education. ^*∗∗*^In case of MIP, the comparison is between *P. vivax* versus *P. falciparum* and mix infection; ^*∗*^ *P. falciparum* + mix infection, ^¥^ *P. vivax*.
