# Supplementary material for: Prevalence of Malaria Infection and Risk Factors Associated with Anaemia among Pregnant Women in Semiurban Community of Hazaribag, Jharkhand, India
Source: Biomed Res Int. 2015 Oct 13;2015:740512. doi: 10.1155/2015/740512 (PMC4691455; doi:10.1155/2015/740512)
Supplement: Supplementary file 1 — Extensive malarial epidemiology related to study sites mentioned in methodology section, detailed demographic information about the subjects investigated during this study as part of method section of manuscript text and some additional and supportive findings mentioned in the results section, which further consolidate and substantiate our observation of asymptomatic prevalence of malaria anaemia in the investigated region has been given in Supplementary material attached to this article. [file 740512.f1.doc]

**Legend Supplementary Table-1**

Baseline characteristics of pregnant women attending antenatal and delivery units

**Legend Supplementary Table 2**

Use of malaria prevention measures by pregnant women attending antenatal clinics and delivery units

**Legend Supplementary Figure-1A-B**

1. Atmospheric temperature prevalence in relation to conduciveness for vector’s growth
2. Correlation between malaria prevalence and temperature of Hazaribag

**Legend Supplementary Figure-2A-C**

1. Transmission Pattern of Malaria (values in number above each bar and below is the year) in Hazaribag (2010-2012).
2. Changes in malaria indices (values in number above each bar and below is the year) in Hazaribag (2010-2012).
3. Monthly and annual percent prevalence profile of malaria in Hazaribag for the year 2012.

**Legend Supplementary Figure-3**

Schematic Flow Chart Summarizing the Sampling Strategy and Groups

**Supplementary** **Table-1**

| **Characteristics** | **Antenatal Clinics**  **N=1271**  N, (%) | **Delivery Units**  **N=870**  N, (%) |
| --- | --- | --- |
| Age (Years) |  |  |
| ˂20 | 166(13.1) | 109(12.5) |
| 20-34 | 98(77.4) | 708(81.4) |
| ≥35 | 122(9.5) | 53(6.1) |
| Prior pregnancies |  |  |
| Primigravid | 423(33.3) | 338(38.38) |
| Secundigravid | 578(45.5) | 209(24.1) |
| Multigravid^*^ | 270(21.2) | 323(37.1) |
| Gestational age at enrollment (weeks)^**^ |  |  |
| ˂20 weeks | 567(44.6) | n/a |
| 20-36 weeks | 641(50.4) | 57(6.5) |
| ≥37 weeks | 63(5) | 813(93.5) |
| Caste |  |  |
| Schedule caste | 169(13.3) | 93(10.7) |
| General caste | 428(33.7) | 307(35.3) |
| Other backward caste | 311(24.5) | 219(25.2) |
| Scheduled tribe | 363(28.5) | 251(28.8) |
| Education |  |  |
| No formal schooling | 357(28.1) | 321(36.9) |
| Attended school any length of time | 914(71.9) | 549(28.8) |
| Socioeconomic characteristics |  |  |
| Owns TV | 567(44.6) | 387(44.5) |
| Owns bicycle | 1173(92.2) | 687(78.9) |
| Owns house | 958(75.4) | 643(73.9) |
| Owns refrigerated | 123(9.6) | 83(905) |
| Roof material |  |  |
| Mud | 622(48.9) | 513(58.9) |
| Corrugated iron/asbestos sheet | 242(19) | 182(20.9) |
| Cement/concrete | 329(25.8) | 107(12.3) |
| Other | 78(6.1) | 68(7.8) |
| Wall material |  |  |
| Mud/sand/dung | 673(52.9) | 478(54.9) |
| Mud bricks | 127(9.9) | 93(10.7) |
| Cement bricks | 419(32.9) | 267(30.7) |
| Other | 52(4.1) | 32(3.7) |
| Primary cooking fuel |  |  |
| Wood | 619(48.7) | 387(44.5) |
| Charcoal | 437(34.4) | 279(32.1) |
| Gas | 153(12.1) | 136(15.6) |
| Other | 62(4.9) | 68(7.8) |
| Mode of delivery among pregnant women |  |  |
| Normal | n/a | 586(67.3) |
| Caesarean | n/a | 179(20.6) |
| Still Birth | n/a | 105(12.1) |
| Birth Outcome |  |  |
| Pre-term delivery(36≤ weeks) | n/a | 129(14.8) |
| Term delivery (31-41 weeks) | n/a | 623(71.6) |
| Post-term delivery (after 41 weeks) | n/a | 118(13.5) |

†Numbers may not add to sample size secondary to missing data.

* Defined as 3 or more pregnancies

** Gestational age assessed by fundal height.

**Supplementary Table 2**

| **Prevention measures utilized** | **Antenatal clinics n=1271  N (%)** | **Delivery units n=870 N (%)** |
| --- | --- | --- |
| Bed net in household | 937(73.7) | 643(73.9) |
| Insecticide-treated bed net in household | 43(3.3) | 21(2.4) |
| Sleeps under net most nights | 873(68.6) | 503(57.8) |
| Taken malaria prophylaxis in pregnancy | 9(0.7) | 3(0.3) |

**Supplementary Figure-1A**

**
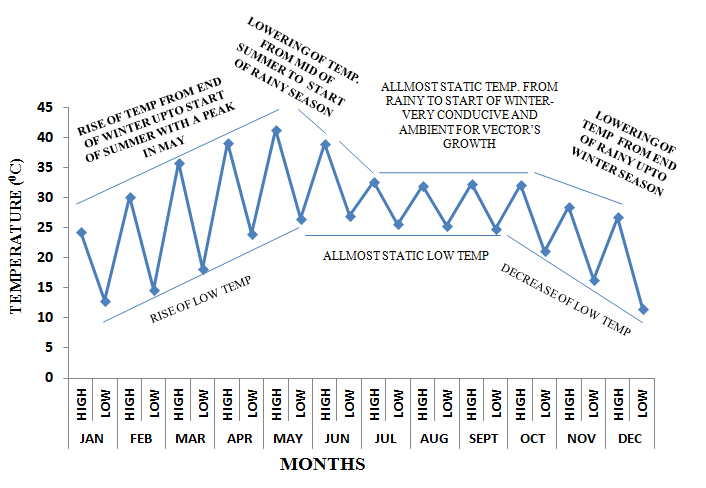
**


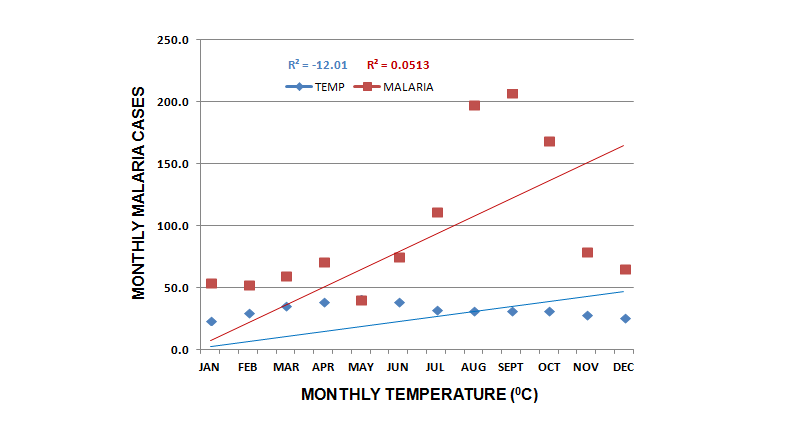
**Supplementary Figure-1B**

**
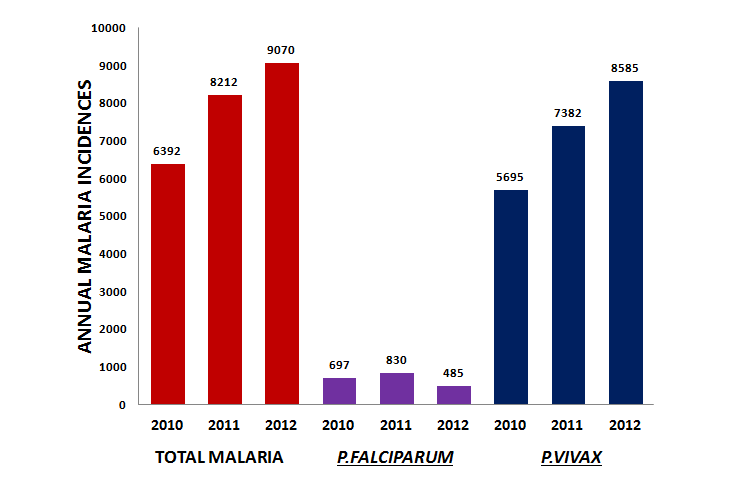
Supplementary Figure-2A**

**Supplementary Figure-2B**


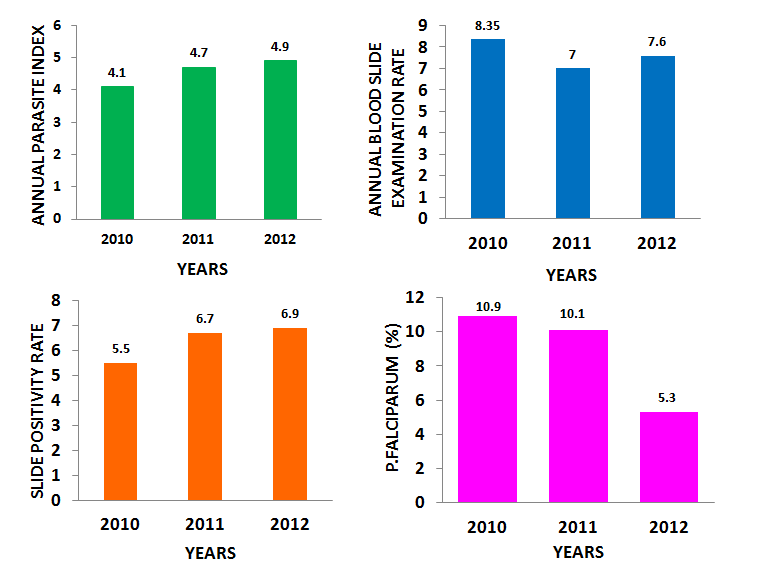


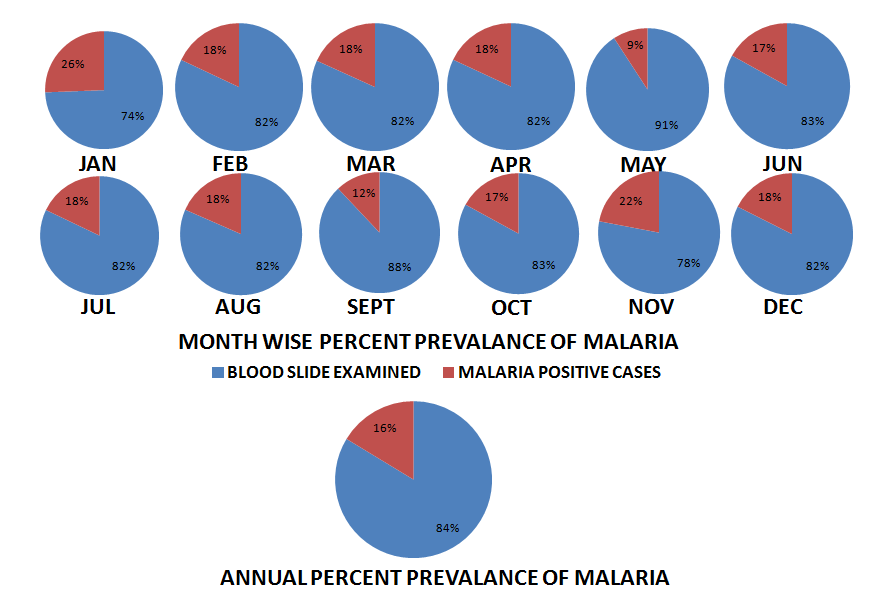
**Supplementary Figure-2C**

**Supplementary Figure-3**

**
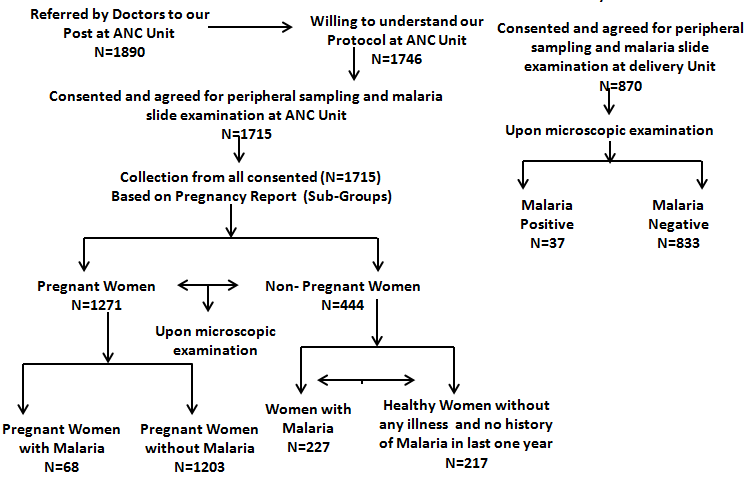
**
